# New insight into rheology and flow properties of complex fluids with Doppler optical coherence tomography

**DOI:** 10.3389/fchem.2014.00027

**Published:** 2014-05-19

**Authors:** Sanna Haavisto, Antti I. Koponen, Juha Salmela

**Affiliations:** Fibres and Biobased Materials, Rheology and Process Flows, VTT Technical Research Centre of FinlandJyväskylä, Finland

**Keywords:** optical coherence tomography, complex fluids, rheology, multi-component fluids

## Abstract

Flow properties of complex fluids such as colloidal suspensions, polymer solutions, fiber suspensions and blood have a vital function in many technological applications and biological systems. Yet, the basic knowledge on their properties is inadequate for many practical purposes. One important reason for this has been the lack of effective experimental methods that would allow detailed study of the flow behavior of especially opaque multi-phase fluids. Optical Coherence Tomography (OCT) is an emerging technique capable of simultaneous measurement of the internal structure and motion of most opaque materials, with resolution in the micrometer scale and measurement frequency up to 100 kHz. This mini-review will examine the recent results on the use of Doppler-OCT in the context of flows and rheological properties of complex fluids outside biomedical field.

## Introduction

Complex fluids are typically composed of several non-homogeneously mixed components. These fluids are often homogeneous at macroscopic scales but disordered at microscopic scales and possess structures of mesoscopic length scales, which play a key role in determining the usually quite intricate properties of the fluid. Some examples of such length scales are the radii of micro emulsion drops, the dimensions of particles in liquid-particle suspension and bubble size in aerogels and foams. Synthetic and biopolymer solutions are one of the most important and widespread class of complex fluids but most multi-phase fluids have these properties. Although the importance of complex fluids is high and yet increasing, the basic knowledge on their properties is inadequate for many practical purposes. This is mainly because of two reasons. Firstly, the variation of structural, rheological, and optical properties of such fluids is extensive and makes it difficult to characterize and categorize them. Secondly, up to the very recent years, there has been lack of effective experimental methods that would allow detailed study of the flow dynamics and structure of especially opaque complex fluids (Gelbart and BenShaul, 1996; Barrat et al., 2005).

The rheology of simple fluids is rather straightforward and well understood. Flow behavior can be characterized with either a single temperature dependent coefficient of viscosity (Newtonian fluids) or with relatively simple relations between the stress and the strain rate (non-Newtonian fluids). Furthermore, these material properties can be accurately measured using conventional rheological methods. It is noteworthy, that the same methods are widely used also in studying the rheological properties of complex fluids. However, acquiring rheological data with complex fluids is not straightforward due to non-uniform and inconstant behavior such as apparent wall slip and wall depletion (Barnes, 1995), particle migration (Leighton and Acrivos, 1987) and shear banding (Olmsted, 2008) that can arise during the experiment. Mechanisms underlying these complicated and poorly understood phenomena are related to the presence of the mesoscopic length scales and its consequences on boundary layer flow (Stickel and Powell, 2005). Fortunately, *local rheological measurements* can be used to overcome these problems.

The local rheological measurements are based on *velocity profiling*. This allows replacing an assumed velocity field by an actually measured profile in analyzing the data for rheological properties of the fluid. The local values of viscosity can be calculated as

(1) $$\mu (y)=\tau (y)\text{/}\dot{\gamma }(y),$$

where $\dot{\gamma }$(*y*) is the shear rate profile (local shear rate) derived from the measured velocity profile and τ(*y*) is the local shear stress obtained either from a pressure difference measurement (capillary or pipe flow geometry) or from a torque measurement (rotational rheometer). The local rheological measurements have captured some of the perturbative effects previously encountered in rheological experiments. For example, both structural probing and velocimetry techniques to detect shear-banded flows have been reviewed in (Manneville, 2008).

Complex fluids are typically highly scattering materials and thus optically turbid. Among the various traditional velocimetry techniques, only the Nuclear Magnetic Resonance Imaging (NMRI) and the Pulsed Ultrasound Doppler Velocimetry (PUDV) are capable of measuring the velocity field (and structure) of such (opaque and heterogeneous) fluids. Both PUDV and NMRI have been tested for several complex fluid and multiphase systems in tube flow and Couette geometry (Seymour et al., 1993, 1995; Britton and Callaghan, 1997; Raynaud et al., 2002; Ovarlez et al., 2011). Especially with tube flows the velocimetry concept is well established and has been implemented as in-line rheometers into industrial processes. These methods have provided novel means for process monitoring and quality control (Arola et al., 1997; Wunderlich and Brunn, 1999; Wiklund and Stading, 2008). However, due to the limited spatial resolution of 40–50 μm (Manneville, 2008) and with PUDV disturbances caused by the wall-fluid interface (Messer and Aidun, 2009), NMRI and PUDV are hardly accurate enough to resolve the flow profile in small flow geometries or in the immediate vicinity of solid boundaries, where e.g., apparent slip can occur.

Optical coherence tomography (OCT) has been developed rapidly in the past years. The use of OCT has been extended from its original application area, biomedicine, to many other fields. A review of OCT applications outside biomedical field (Stifter, 2007) presents several application cases from dimensional metrology and materials research to data storage and security applications. OCT has been introduced also for velocity profiling (Chen et al., 1997) but somewhat surprisingly, the potential of OCT in rheological applications has only very recently been realized (Harvey and Waigh, 2011; Lauri et al., 2011).

In this article we review some of the recent developments and applications of OCT outside of medical fields. We focus our review to the use of OCT to measure flow properties and rheology of complex fluids. At first, a very brief summary of the origin and basic principle of OCT with emphasis on velocity measurement (Doppler-OCT) is given. Finally, some recent promising studies are presented.

## Principle of optical coherence tomography

### Origin and methods

OCT was introduced in the early nineties for non-invasive depth-resolved imaging with micrometer resolution. Penetration depth depends greatly on optical properties of the material and used OCT principle. Penetration depth varies from micrometers to millimeters. The principle of OCT imaging is analogous to ultrasound, except that it utilizes light instead of acoustic waves, thereby achieving image resolutions of one to two orders of magnitude higher than standard ultrasound (Fujimoto, 2003). The functional principle of OCT is based on interpretation of the interference patterns of white or low-coherence light emitted to the sample. The interference pattern contains simultaneous information of the location, scattering index (density) and the travelling speed of the scattering particles (Stifter, 2007). In practice, OCT imaging is realized using a Michelson interferometer with a low-coherence-length light source, such as super luminescence diodes or solid state lasers (Fujimoto, 2003).

There are two main OCT methods that are classified based on the interferometric technique. In time domain OCT (TD-OCT) the measurement volume is moved through the sample by changing the position of a reference mirror and the scattering interference is measured sequentially for all locations. The other option is frequency or spectral domain OCT (FD-OCT or SD-OCT) where the whole imaging depth is captured instantaneously and no reference mirror movement is required. In this case the interference pattern is divided spectrally to different detectors and information from different measurement depths is analyzed simultaneously. For the physical fundamentals as well as comparative analysis on the OCT methods the reader is encouraged to refer to review articles or textbooks (e.g., Schmitt, 1999; Fercher et al., 2003; Brezinski, 2006; Drexler and Fujimoto, 2008).

In addition to cross-sectional tomography imaging OCT was soon adopted for measuring flow velocities. Coherence gating concept for measuring velocity profile of water flow in a duct was introduced in 1991 (Gusmeroli and Martinelli, 1991). The extension of OCT to measure flow velocities is called Doppler-OCT (D-OCT) or Optical Doppler Tomography (ODT). The Doppler frequency generated by moving objects is detected from the phase difference of two adjacent OCT measurements and it is proportional to the sample velocity. The velocity component parallel to the incident beam generates a Doppler shift to the signal and this that can be detected with a single component OCT system.

### Velocimetry with doppler optical coherence tomography (D-OCT)

After the basis of D-OCT velocity profiling was established the method has been further developed for advanced velocimetry. D-OCT velocity profiling has been validated against laminar flow of microparticle suspensions in cylindrical and square conduits either in air or in conduits submerged in highly scattering media mimicking tissue (Wang et al., 1995, 1997; Chen et al., 1997). For fully developed pipe flow D-OCT velocity profile measurement can quantify the average flow but for this accurate determination of the Doppler angle is crucial (Drexler and Fujimoto, 2008). Thus, methods for accurate determination of the Doppler angle and velocities perpendicular to probe beam have been developed. These methods have improved possibilities to investigate flows in complex geometries or within biological systems where evaluation of the Doppler angle is unfeasible (see e.g., Piao and Zhu, 2003; Wu, 2004; Wang and Wang, 2010).

The most well-known target of D-OCT measurements has been the flow of blood in real and artificial blood vessels. Velocity mapping with D-OCT has been performed in different locations within sudden contraction (Proskurin et al., 2003, 2004) and T- and Y-shaped vessel junctions (Bonesi et al., 2007a,b, 2010). More recently, D-OCT has been used to investigate capillary-driven blood flow (Cito et al., 2012).

Besides the intense attention in measuring blood flow, the potential of OCT for many other fluid dynamical applications have been reported. Applications covering this topic have been reviewed thoroughly in Refs. (Brezinski, 2006; Stifter, 2007; Drexler and Fujimoto, 2008). We mention here only flows in micro-scale geometries that provide a natural and successful platform for D-OCT. Examples on microchannel flows can be found in Wang et al. (2004), Marks et al. (2004), Ahn et al. (2005), Lauri et al. (2008) and for micron-scale boundary layer flows in Kempe et al. (2007), Mujat et al. (2013), and Gao et al. (2013).

## Application of D-OCT for measuring rheology of complex fluids

D-OCT is a very promising tool for studying rheology in capillaries (Lauri et al., 2011), in rotational rheometers (Harvey and Waigh, 2011) and in elongational rheology (Dufour et al., 2005). By combining a rotational rheometer with D-OCT simultaneous measurement of the real velocity profile across the rheometer gap together with the shear stress has been reported (Harvey and Waigh, 2011; Jaradat et al., 2012; Saarinen et al., 2014). When the size of the flow geometry exceeds the maximum measuring distance of OCT the measurements can be complemented by measuring the outer flow velocity profile with e.g., PUDV or MRI techniques (Salmela et al., 2013). Combining such data with the structural information opens truly unprecedented research prospects for the study of rheology of opaque fluids.

**Figure 1:**
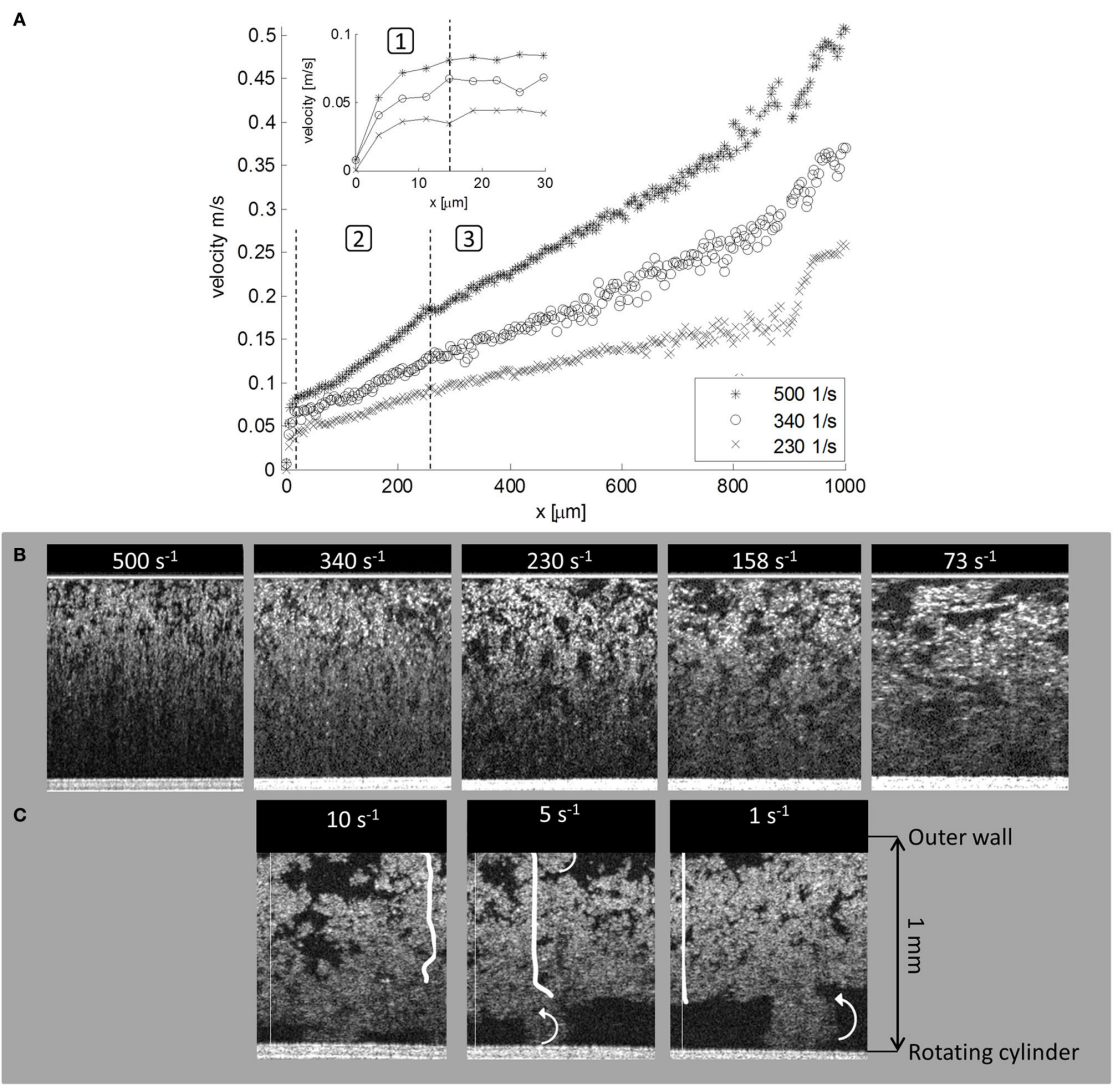
**OCT combined with a rotational rheometer equipped with smooth concentric cylinders geometry. (A)** At high apparent shear rates the velocity profiles of 1% MFC have three regions: (1) Wall slip layer with thickness of ~15 μm. (2) Wall boundary layer (~250 μm) where the slope of the velocity profile is higher than in the middle of the gap. (3) The outer layer, where the shear rates are 55–70% smaller than the apparent shear rate. Close to the moving boundary OCT profiles are distorted because the intensity of the scattering signal becomes too weak. **(B)** MFC suspension structure at moderate and high apparent shear rates. **(C)** Structure and velocity profiles for small apparent shear rates. From left to right: Complete slip on the stationary wall, slip and rolling flocs on both walls, and rolling flocs on the moving wall. Original figures are presented in Haavisto et al. (2014).

One interesting material studied in this context is microfibrillated cellulose (MFC). MFC is produced from cellulose fibers using mechanical, chemical, or enzymatic disintegration methods. These fibrils have very high specific surface area, aspect ratio, strength, and flexibility. MFC fibers generally form an opaque strongly flocculated suspension with high viscosity and yield stress already at low mass concentrations (Pääkkö et al., 2007; Lavoine et al., 2012). These properties make the usage of most flow analysis methods very difficult. Rheology of MFC suspensions has often been studied by interpreting shear viscosity from flow curve measurements (Agoda-Tandjawa et al., 2010; Iotti et al., 2011; Karppinen et al., 2012). Based on the flow curve MFC behaves like a strongly shear-thinning power law fluid (μ ~ ${\dot{\gamma }}^{n}$) with a Newtonian plateau between the low and high shear rate regions. A rheometer augmented with OCT reveals whether the flow curve actually represents true material properties of the suspension or if the flow curve is merely reflecting boundary layer phenomena like apparent wall slip (Haavisto et al., 2014; Saarinen et al., 2014). From the structure and velocity data the factors interfering with the rheological results are rather obvious (Figure 1). These results indicate that the MFC shear viscosity measured using rotational rheometer corresponds to suspension material properties only at high shear rates (after the Newtonian plateau). Even at the high shear rates three distinguished shear rate regions have been identified: Wall slip layer, wall layer and outer layer. In all the regions the velocity profiles differ from the ideal (assumed) one, which introduces some error to the apparent shear rate values.

Similarly as for MFC, non-ideal velocity profiles in rheometer gap have been demonstrated for margarine and for polymer solutions (Harvey and Waigh, 2011; Jaradat et al., 2012). Experiments performed with OCT rheometry captured shear-banding and wall slip in semi-dilute polyacrylamide (PAM) solutions when the molecular weight and the concentration of the polymer in the solution were high enough.

**Figure 2:**
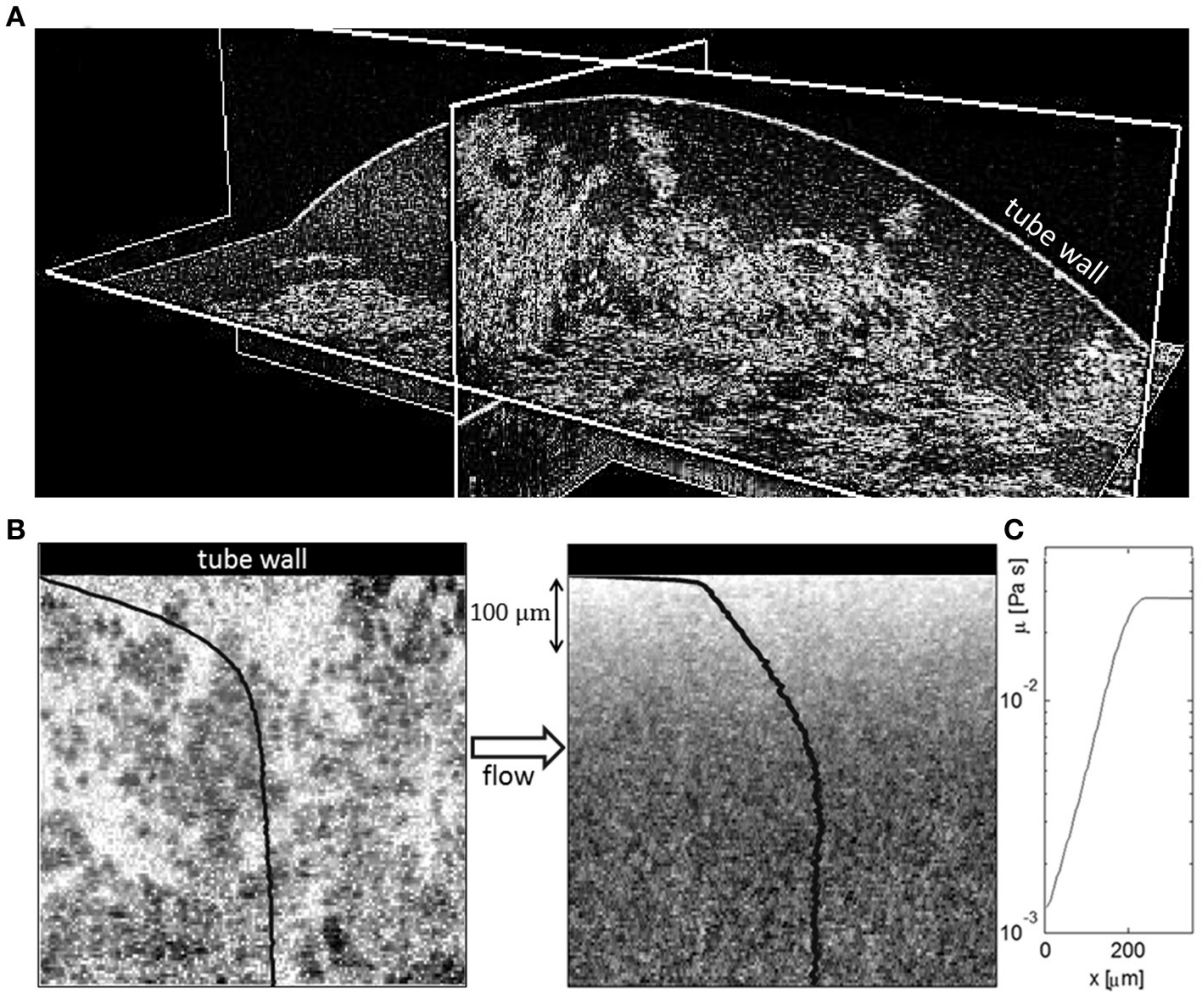
**(A)** 3D OCT image of MFC suspension in a pipe with diameter of 8.6 mm. Fiber-poor regions are clearly visible close to the pipe wall (curved surface). **(B)** 2D OCT image of flowing MFC (0.4%) suspension near the tube wall. Shown are an instantaneous image (left) and an average of 200 independent images (right) acquired with SD-OCT. The gray-scale values in the image represent the local value of the optical back-scattering index, light color corresponding to low index value. The average velocity profile and the average scattering intensity i.e., effective concentration of the suspension are shown with black curves; the velocity profile on the left image and effective concentration on the right image. **(C)** The calculated viscosity profile close to the wall. Original figures are presented in Salmela et al. (2013) and Haavisto et al. (2014).

The mechanisms behind the non-ideal velocity profiles have been discussed by Salmela et al. (2013), Haavisto et al. (2014). OCT data has been analyzed separately for the structure information and for the local viscosity information. They report an almost fiber free layer very close to the pipe wall (Figure 2). In this layer the locally calculated viscosity is very close to that of water. With increasing distance the viscosity increases exponentially until it saturates to its bulk value approximately at 200 μm from the wall. Contribution of this apparent slip layer may be as much as 95% to the total volumetric flow rate (Haavisto et al., 2014).

## Conclusion

OCT activities are presently dominated by various biomedical applications, but we expect that the use of OCT will spread rapidly to numerous other fields. As the D-OCT method is capable of accurate high-resolution detection of simultaneous velocity and structure measurements it is exceptionally well suited in detailed study of the flow behavior and rheology of complex fluids in microfluidic devices or close to solid boundaries. Besides D-OCT there are many other OCT methods that are potentially very useful for rheological studies: Polarization sensitive OCT can be used for the analysis of particle and polymer orientation (Stifter et al., 2003), ultra-high resolution OCT (UH-OCT) will facilitate detailed understanding of the flow and structure down to sub-micron length-scales (Czajkowski et al., 2011), and dynamic light scattering OCT (DLS-OCT) can be utilized to obtain two or even three velocity components simultaneously (Huang and Choma, 2014). In the near future we expect to see more industrial applications where the OCT and PUDV (or MRI) techniques have been combined to study the real rheological properties of complex fluids. OCT will also give totally new insight in to the deformation of material in rheometer systems.

### Conflict of interest statement

The authors declare that the research was conducted in the absence of any commercial or financial relationships that could be construed as a potential conflict of interest.
